# Pesticide residues in honeybee-collected pollen: does the EU regulation protect honeybees from pesticides?

**DOI:** 10.1007/s11356-021-16947-z

**Published:** 2021-10-23

**Authors:** Lotta Kaila, Jarmo Ketola, Marjaana Toivonen, Olli Loukola, Kati Hakala, Sakari Raiskio, Timo Hurme, Marja Jalli

**Affiliations:** 1 Department of Agricultural Sciences, University of Helsinki, P.O. Box 27, 00014 Helsinki, Finland; 2 Natural Resources Institute Finland (Luke), Latokartanonkaari 9, 00790 Helsinki, Finland; 3 Natural Resources Institute Finland (Luke), Tietotie 4, 31600 Jokioinen, Finland; 4 Finnish Environment Institute (SYKE), Biodiversity Centre, Latokartanonkaari 11, 00790 Helsinki, Finland; 5 Ecology and Genetics Research Unit, University of Oulu, University of Oulu, PO Box 3000, 90014 Oulu, Finland; 6 Finnish Food Authority, Mustialankatu 3, 00790 Helsinki, Finland

**Keywords:** Sustainable agriculture, Pollinators, Field-realistic residues, EU legislation, Implementation, Risk assessment

## Abstract

Researchers globally identify pesticides as one of the main reasons for pollinator decline. In the European Union (EU), extensive legislation is implemented to protect pollinators from harmful pesticide exposure. The aim of our study was to discover whether the pesticide residue levels in honeybee matrices, such as nectar and pollen, exceeded the chronic or acute toxicity levels when beehives were located next to fields treated with specific insecticides. The insecticides were used according to the EU legislation and its national implementation. The experiments were conducted in turnip rape, oilseed rape, and caraway fields in southern Finland during the years 2019 and 2020. The pesticides used in the experiments contained the active substances lambda-cyhalothrin (2019), esfenvalerate (2020), and tau-fluvalinate (2020). However, the honeybee-collected pollen and nectar were analyzed for residues of more than 100 active substances. The results showed that the pesticide residue levels clearly remained under the oral acute toxicity for honeybees, although we found high levels of thiacloprid residues in the pollen collected in 2019. The pesticide residues in nectar were below LOQ values, which was most likely due to the rainy weather conditions together with the chosen sampling method. No statistically significant differences were observed between the insecticide-treated and untreated fields. In light of our research, the EU legislation protected honeybees from oral acute toxicity during the years 2019 and 2020. However, potential sublethal effects of thiacloprid and other pesticide compounds found in the collected pollen cannot be ruled out. In the future, constant monitoring of pesticide exposure of honeybees and wild pollinators should be established to ensure that pesticide legislation, and its implementation across the EU successfully protects pollinators and their services in agricultural environments.

## Introduction

Pollinator decline causes concern globally (Potts et al. 2010). Insect pollination plays a major role in agricultural crop production, and the pollinator decline has major food security consequences (Aizen et al. 2008). The intergovernmental science-policy platform on biodiversity and ecosystem services (IPBES) identifies pesticides (i.e., plant protection products), particularly insecticides, as one of the main reasons for pollinator decline (IPBES 2016). Insecticides protect crop plants by killing plant-feeding insects that may reduce the crop yield, but may be harmful for beneficial insect species such as pollinators (Brittain & Potts 2011).

Pollinators are exposed to pesticides through several routes (Benuszak et al. 2017), with the most critical oral exposure routes being pollen and nectar (Zioga et al. 2020). The oral exposure may take place when pollinator-attracting crops are treated with pesticides. Oilseed rape (*Brassica napus* subsp. *oleifera*) and turnip rape (*Brassica rapa* subsp. *oleifera*) are examples of crops that strongly attract honeybees and wild bees but also suffer from harmful pests such as common pollen beetle (*Brassicogethes aeneus*) that may destroy the entire yield if not controlled (e.g., Toivonen et al. 2019; Lindstöm et al., 2017). Commonly used insecticides in the cultivation of oilseed brassicas include pyrethroids tau-fluvalinate, esfenvalerate, and lambda-cyhalothrin, as well as the nenonicotinoid thiacloprid, which all interfere with the insect nerve system (Davies et al. 2007; Tomizawa & Casida 2005). Insecticide treatment at an early flowering stage may increase the risk for bee pesticide exposure because the crop may attract bees soon after treatment.

The European Union (EU) Regulation (EC) No. 1107/2009 states that the use of pesticides in the EU should not represent an unacceptable acute or chronic risk to honeybees or unacceptable effects on honeybee colony survival and development. The regulation requires several steps for the approval of an active substance used in a pesticide, the most crucial steps for the honeybees being the following three: (1) pesticide companies provide studies on the effects of the active substance on honeybees and if needed, on residues in nectar and pollen; (2) one reporter Member State (MS) assesses the delivered data; and (3) the European Food Safety Authority (EFSA) together with MSs peer reviews the active substance assessment. Products containing active substances are assessed and authorized separately at the MS level, where approved application rates and potential risk mitigation measures are determined.

Despite the broad risk assessment scheme aimed at protecting honeybees, pesticides have caused damage to pollinators in the EU. For example, Rundlöf et al. (2015) found that the insecticides clothianidin and β-cyfluthrin reduced wild bee density, solitary bee nesting, and bumblebee colony growth and reproduction in a field experiment in Sweden. Another insecticide, dimethoate, caused severe honeybee losses to beekeepers in 2015 and 2017 in western Finland (Finnish Safety and Chemicals Agency, Personal communication). However, most of our knowledge on insecticide effects on bees comes from laboratory and semi-field experiments, whereas the actual insecticide exposure under field conditions remains relatively little studied (Godfray et al. 2014, 2015). Zioga et al. (2020) concluded in their review that the research on pesticide residues in pollen and nectar covers only a fraction of the global area and focuses mainly on a single pesticide group, neonicotinoids. In Finland, for example, only one study with a focus on neonicotinoids has examined honeybee pesticide exposure (Ketola et al. 2015). Large variation across time and geographical location in pesticide residue levels can be expected because pesticide exposure is affected by environmental conditions and agricultural practices (Zioga et al. 2020; Böhme et al. 2018). To study field-realistic pesticide residues and their variation in honeybee-collected pollen, Böhme et al. (2018) analyzed pesticide residues in southern German apiaries. In the 5-year study, the researchers showed that the pesticide range, concentration, and combinations in the pollen varied depending on the agricultural intensity at the study site, study year, and time of the growing season. This highlights the need for multi-year and multi-site studies to obtain a comprehensive picture of field-realistic pesticide exposure of pollinators.

Our research examined how the implementation of the EU Regulation ((EC) No. 1107/2009) protected honeybees (*Apis mellifera*) from pesticide exposure, by studying pesticide levels in honeybee-collected pollen and nectar in southern Finland. The aim was to study whether the residue levels remained below honeybee oral acute and chronic toxicity levels that are used in the pesticide risk assessment process in the EU. The beehives were located next to mass-flowering caraway (*Carum carvi*), oilseed rape and turnip rape fields that were treated with specific pesticides according to the EU legislation and its national implementation.

## Methods

### Study fields

The experiments were conducted in 2019 and 2020 in southern Finland in the adjacent municipalities of Jokioinen and Ypäjä. The agricultural area of these municipalities totals about 14, 000 ha, and during the years 2019 and 2020, cereals and grassland represented 60% and 30% of this area, respectively. Oilseed rape, turnip rape, and caraway were minor crops in the area, oilseed rape, and turnip rape together covering 2% and caraway ≤ 1.5% of the agricultural area in 2019–2020 (Loimaan maaseutupalvelu, Personal communication).

**Fig. 1:**
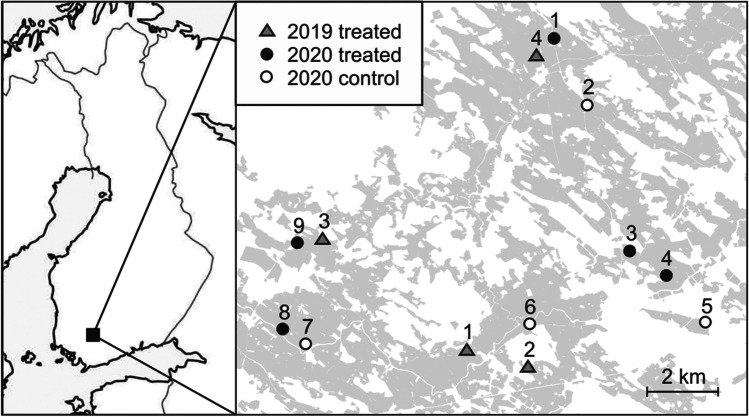
Locations of the study fields in southern Finland in 2019 and 2020. Arable land is shown on the map in gray

In 2019, we conducted a preliminary study in the fields of local farmers. The aim was to establish best practices for conducting a more extensive experiment the following year. The experiment in 2019 was carried out in two caraway fields (each about 8 ha), one spring turnip rape field (1.2 ha), and one winter oilseed rape field (6 ha) (Table 1). The three crops were selected because they attract honeybees. The fields were 2–8.5 km apart (Fig. 1). A pesticide containing the active substance lambda-cyhalothrin was used in all the fields (Table 2). Lambda-cyhalothrin was chosen for the study because the substance is used in Finland in caraway, oilseed rape, and turnip rape at an early flowering stage, which increases the risk of honeybees to be exposed to the substance. In 2020, the main experiment took place in nine spring oilseed rape fields on land of the Natural Research Institute Finland (Fig. 1, Table 1). Five of the fields were treated with pesticides containing the active substances esfenvalerate and tau-fluvalinate (Table 2), while four fields remained as untreated controls. Esfenvalerate and tau-fluvalinate were chosen for the study for the same reason as lambda-cyhalothrin in 2019: the substances are used at early flowering stages, when foraging bees can become exposed to them. All the fields were approximately one hectare in size, and they were located 0.7–11 km apart (Fig. 1). Although honeybees can forage up to several kilometers from their hives, they prefer nearby resources when available (Couvillon et al. 2015). Since the study fields were at similar growing stage, the bees were likely to choose the field closest to their hive. In both study years, cultivation methods respected the instructions for pesticide use that the national authority authorized for the commercial products (Table 2). Just prior to the first pesticide treatment, two equally strong beehives (in 2019) or one beehive (in 2020) were placed within 5 m of each study field, according to common practice in Finland (Table 1). The honeybee colonies were maintained in ten-frame Farrar hives with 5–6 brood frames, 2–3 frames with food reserves (pollen and honey), and 1–2 empty frames. The number of beehives per field and the size of the study fields were smaller in 2020 in order to maximize the number of study locations. The number of beehives followed the recommendation of the Finnish beekeepers’ association to have one beehive per one hectare of oilseed rape.

**Table 1:** The study fields, crops, cultivars, sowing dates, and the dates of placing beehives next to the study fields

Field	Crop	Cultivar	Sowing date	Beehives placed on the field margins (date)
Study year 2019				
1	Winter oilseed rape	Dk Sequoia	02/08/2018	19/05/2019
2	Caraway	Record	03/06/2017	03/06/2019
3	Caraway	Prochan	21/06/2017	01/06/2019
4	Turnip rape	Drago	10/05/2019	23/06/2019
Study year 2020				
1	Spring oilseed rape	Drago	27/05/2020	25/06/2020
2, 4	Spring oilseed rape	Drago	25/05/2020	25/06/2020
3, 5	Spring oilseed rape	Drago	22/05/2020	25/06/2020
6–9	Spring oilseed rape	Drago	23/05/2020	25/06/2020

**Table 2:** Pesticide treatments in the study fields in 2019 and 2020

Field	Crop	Date	Time	Active substance	Product name in Finland	Commercial product application rate ml/ha	Active substance concentration g/l
Study year 2019							
1	Winter oilseed rape	05/20/2019	21–06	Lamda-cyhalothrin	Karate Zeon -tekniikka	37	100
2	Caraway	03/06/2019	21–06	Lamda-cyhalothrin	Karate Zeon -tekniikka	60	100
3	Caraway	03/06/2019–04/06/2019	21–06	Lamda-cyhalothrin	Karate Zeon -tekniikka	60	100
4	Turnip rape	11/06/2019	21–06	Deltamethrin	Decis Mega EW 50	100	50
4	Turnip rape	17/06/2019	06–21	Thiacloprid	Biscaya OD 240	400	240
4	Turnip rape	25/06/2019	21–06	Lamda-cyhalothrin	Karate Zeon -tekniikka	37	100
4	Turnip rape	01/06/2019	21–06	Lamda-cyhalothrin	Karate Zeon -tekniikka	37	100
Study year 2020							
1–9	Spring oilseed rape	seed coating		Flupyridifuron	Buteo Start FS 480	63	480
1–9	Spring oilseed rape	16/06/2020–17/06/2020	21–06	Deltamethrin	Decis Mega EW 50	100	50
1–9	Spring oilseed rape	23/06/2020–24/06/2020	21–06	Lamda-cyhalothrin	Karate Zeon -tekniikka	37	100
1, 3–4, 8–9	Spring oilseed rape	27/06/2020–28/06/2020	21–06	Esfenvalerate	Sumi alpha 5 FW	150	50
1, 3–4, 8–9	Spring oilseed rape	05/07/2020	06–21	Tau-fluvalinate	Mavrik	200	240
1, 3–4, 8–9	Spring oilseed rape	10/07/2020	06–21	Tau-fluvalinate	Mavrik	200	240

Both in 2019 and 2020, the pest control thresholds set in the label text of the commercial products defined the pesticide treatments. Turnip rape and oilseed rape were treated if at least one *Phyllotreta undulata* or *P. striolata* appeared per plant at cotyledon stage, or if three to four *B. aeneus* appeared per plant before flowering. The threshold for caraway was reached if individuals of *Depressaria daucella* appeared on sticky traps in the field. The development stage of the crop plants was taken into account in the timing of the pesticide treatments: tau-fluvalinate was spread before the seedlings exceeded BBCH stage 60 (first flowers open), and lambda-cyhalothrin and esfenvalerate before the seedling exceeded BBCH stage 59 (first petals visible, flower buds still closed) (Federal Biological Research Centre for Agriculture and Forestry, 2001).

### Pollen and nectar sampling

Pollen samples were collected in 2019 (23 samples) and in 2020 (41 samples). Pollen traps were installed in the beehives before the first and immediately after every pesticide treatment. The aim was to collect pollen 24 h after each treatment, but in practice, the collection time was 2–24 h due to rainy weather (Table 4 in the Appendix). Nectar was collected from the hives only in 2020. The nectar samples (23 samples) of minimum 3 g per sample were collected from the honey cones inside the beehives 8–24 h after each treatment except the last tau-fluvalinate treatment. The aim was to sample fresh, newly collected nectar that the honeybees had collected in the sampling day. After the last treatment, the bees did not collect enough nectar for sampling. All samples were stored at – 20 °C within an hour from sampling.

### Pesticide multi-residue analysis

Pollen samples were analyzed for 108 active substances in 2019. Pollen and nectar were analyzed for 110 active substances in 2020 (Table 6 in the Appendix). The analyzed active substances were not optimized for this study, but the substances include in a routine pesticide scope of laboratory for beehive products. At the beginning of the study, it was ensured that the pesticides used in the studied fields are monitored by the multi-residue method. Sample pretreatment methods were based on a modified Quenchers method (Anastassiades et al. 2003). (1) Three grams of nectar was weighed to the analyses. Salting out was proceeded with water (6.5 ml), acetonitrile (10 ml) and Supel™ QuE Citrate extraction mixture. Six milliliter of acetonitrile phase extract was purified with the dispersive SPE (Supel™ QuE PSA/C18). 2) Pollen was homogenized and 0.5 g was weighed to the analysis. Salting out and liquid–liquid extraction were proceeded with the water (8 ml), hexane (3 ml), and acetonitrile (10 ml) (Supel™ QuE Citrate). Acetonitrile phase was separated and kept in the freezer overnight (18 °C); after that, 6.5 ml was cleaned with the dispersive SPE (Supel™ QuE PSA/C18). Final acetonitrile extract from the pollen and nectar treatments was filtered (0.22 µm GHP) and analyzed straight by liquid chromatography mass spectrometry. For the gas chromatography analysis, part of the extract was evaporated and reconstituted in acetone. Depending on the active substance, the analyses were performed with liquid chromatography tandem mass spectrometry LC–MS/MS (Shimadzu LCMS-8050 and Nexera X2 UHPLC, Shimadzu USA Manufacturing Inc., USA) or gas chromatography tandem mass spectrometry GC–MS/MS (TSQ Quantum XLS Ultra, Trace GC Ultra, Triplus RSH, Thermo Fisher Scientific, USA) with the multiple reaction monitoring (MRM) techniques. Mass spectrometric reaction used for the quantification and device, GC or LC, is shown in Table 6 in the Appendix. Ionization techniques were electrospray (ESI) or electronic (EI). LC column was Kinetex® 2.6 µm Biphenyl 100 Å (2.1 mm × 100 mm), and linear gradient elution with the 10 mM NH_4_Ac and methanol was used. GC column was Phenomenex Zebron ZB-50 (0.25 µm, 30 m × 0.25 mm). Procedural calibration (5 points) was used for the quantification. Quality control and calibration level were different between two study years. Therefore, the limit of quantification (LOQ) was 10–40 µg/kg in 2019, and 0.5–30 µg/kg in 2020, depending on the active substance. Some compounds were identified (Appendix X and X) at the concentration level < LOQ. Those cases, chromatographic peak shape, retention time, and relation of quantifier ion and qualifier ion, were similar than in reference standard sample. In addition, signal to noise relation was enough to reliable separation (> 3) Table 6.

Glyphosate and its metabolite AMPA were analyzed from all nectar samples, and glyphosate from pollen samples taken on June 28th, July 5th, and July 6th. Glyphosate and AMPA were analyzed with the method based on FMOC derivatization and LC–MS/MS (Shimadzu LCMS-8050 and Nexera X2 UHPLC, Shimadzu USA Manufacturing Inc., USA). Column was Acquity UPLC® BEH C18 1.7 µm, 2.1*100 mm and linear gradient with 10 mM NH_4_Ac and acetonitrile was used for the elution. 3 g of the nectar or 1 g of pollen was weighted to the analysis. Residues were extracted into 50 ml or 15 ml of water, respectively. Derivatization reaction (at minimum 2 h) was performed in the reaction mixture containing equal amounts of sample extract, 5% borate buffer, and FMOC solution (10 mg/ml FMOC in ACN). Derivatization solution was filtered (0.22 µm GHP) before LC–MS/MS analysis. Procedural calibration (5 point) and deuterated internal standard were used for the quantification. The limit of quantification for glyphosate and AMPA was 10 µg/kg.

### Pesticide exposure per bee

The pesticide oral exposure of adult honeybees and larvae was calculated based on detected pesticide residues in 2019 and 2020 and the pollen and nectar consumption given in the Bee Guidance Document of the EFSA: a forager bee consumes 0 mg pollen and 32–128 mg sugar from nectar per day; a nurse bee consumes 6.15–12 mg pollen and 34–50 mg sugar from nectar per day; and larvae consume 1.5–2 mg sugar and 59.4 mg sugar from nectar per 5 days (European Food Safety Authority, 2013). The calculations assumed that the oilseed rape nectar contained a maximum of 30% sugar, an average value used in the Bee Guidance Document (European Food Safety Authority 2013); hence, maximum consumption of nectar was 426.67 mg (128 mg/0.3) for a forager, 166.67 mg for a nurse bee, and 198 mg for a larva. The lowest exposure level was counted for each substance by multiplying the lowest detected pesticide residue level in the pollen samples by the lowest estimated pollen consumption by nurse bees and larvae. Respectively, the highest exposure level was counted by multiplying the highest pesticide residue level in the pollen samples by the highest estimated pollen consumption by nurse bees and larvae. The acute toxicity levels for honeybees were defined in EFSA’s conclusions on pesticide peer review of the pesticide risk assessment of the active substance concerned. However, the peer reviews of the studied pesticides covered chronic toxicity level for honeybees only for indoxacarb, and hence, the chronic toxicity of the detected residues could not be estimated for the other active substances found in the study.

### Oilseed and caraway pollen identification

**Fig. 2:**
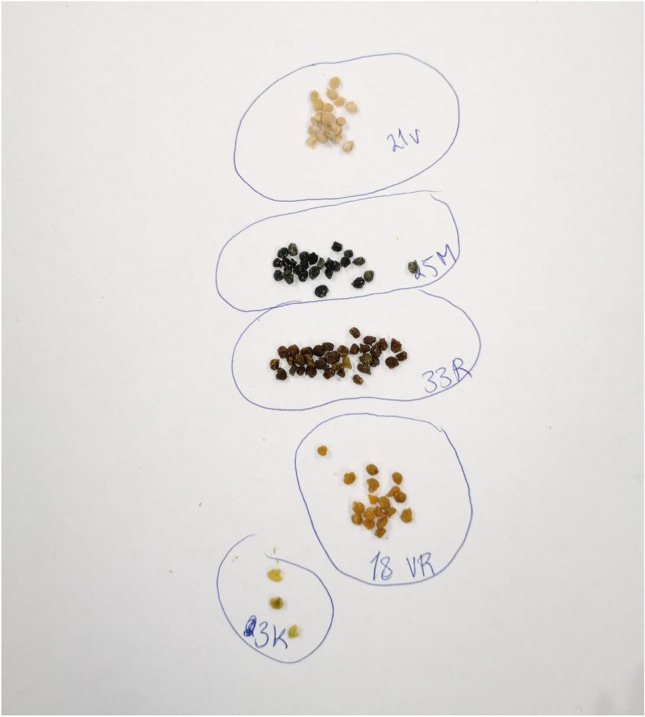
The color difference of identified pollen samples. The pollen is from field 5 taken on 28/06/2020. The sample consists of 21 white pellets (V), 25 black pellets (M), 33 brown pellets (R), 18 light brown pellets, and 3 yellow pellets which we assume to be oilseed rape pollen

The oilseed (oilseed rape or turnip rape) and caraway pollen were identified to get an overview of the percentage of those crops in the pollen samples. In 2019, the identification was done on genus level morphologically with light microscopy and visually based on the color of the pollen. In 2020, the identification was carried out only visually based on the color of the pollen. The visual identification was done based on a color map by the Finnish beekeepers’ association (Suomen Mehiläishoitajain Liitto ry., 2019) so that specific yellow pellets were identified as oilseed rape pollen (Tables 4 and 5 and Fig. 2 in the Appendix).

### Statistical analysis

Only data from 2020 were analyzed statistically (2019 acted as a preliminary study and did not have a specific statistical design). In 2020, the statistical design was a repeated measurements design with four time points, five, cases and four controls. The four time points were (1) residues before any pesticide treatment, (2) residues after the esfenvalerate treatment, (3) residues after the first tau-fluvalinate treatment, and (4) residues after the second tau-fluvalinate treatment. The impacts of the esfenvalerate and tau-fluvalinate treatments on residue levels in pollen (no residues were detected in the nectar) were tested by comparing residues in beehives located next to treated fields and untreated control fields using nonparametric one-way ANOVA (Kruskal–Wallis test) for each timepoint separately. In addition, the relationship between the concentration of oilseed rape pollen in the sample and the tau-fluvalinate residue levels was examined using correlation analysis. Both statistical analyses were conducted with SAS Enterprise Guide 7.1.

## Results

### Weather conditions

Weather conditions differed between the years 2019 and 2020 (Table 7 in the Appendix). In 2019, the weather was sunny at the time of the pesticide treatments, and the crops began blooming quickly. By contrast, in 2020, rainy weather challenged the experiment, leaving only short few rainless periods for pesticide applications and honeybee foraging. Furthermore, the rainy and cold weather slowed the start of crop flowering, and hence, the honeybees did not forage as much oilseed rape pollen after the pesticide treatment as in 2019. The rainfastness of the pesticides, i.e., the required minimum time period from the pesticide treatment to the next rain, was always respected.

## Active substances in the pollen and nectar honeybee oral exposure

In 2019, a total of five active substances were found in the honeybee-collected pollen (hives next to caraway, oilseed rape, and turnip rape fields). Lambda-cyhalothrin, the active substance with which all the fields were treated when the beehives were placed on the field margins, was found in three samples (caraway and oilseed rape) out of 24 analyzed pollen samples: the residue levels ranging from 3.2 to 7.8 µg/kg (Table 3, Table 5 in the Appendix). In addition to lambda-cyhalothrin, four other active substances were found in the pollen, thiacloprid, tebuconazole, azoxystrobin, and indoxacarb, of which the last three ones were not sprayed in the study fields. Tebuconazole and azoxystrobin are fungicides used, for example, in oilseed rape fields during flowering. Indoxacarb is an insecticide used, for example, in oilseed rape fields before flowering. Nectar was not analyzed in 2019.

**Table 3:** Detected residue levels in pollen µg/kg and the calculated oral exposure of nurse bees and larvae (µg/insects) in 2019 and 2020

Active substance	µg/mg × 10^−6^		Exposure µg/nurse bee/day × 10^−6^		Exposure µg/larvae/5 days × 10^−6^		LD50 µg/adult bee × 10^−6^	LC50 (10 days exposure) µg/ adult bee × 10^−6^	How many times bigger LD50 value is than the highest exposure level of a nurse bee?*
Pollen consumption	Lowest	Highest	Lowest (6.5 mg)	Highest (12 mg)	Lowest (1.5 mg)	Highest (2 mg)			
Year 2019									
Azoxystrobin	≈ 3.3	≈3.8	21.5	45.6	5.0	7.6	25 000 000.0	LC50 unknown	548 000
Indoxacarb	≈ 2.5	≈2.5	16.3	30.0	3.8	5.0	232 000.0	64 900.0	8000
Lambda-cyhalothrin	≈ 3.2	≈7.8	20.8	93.6	4.8	15.6	910 000.0	LC50 unknown n	10 000
Tebuconazole	≈ 0.3	≈1.3	2.0	15.6	0.5	2.6	83 050 000.0	LC50 unknown	5 324 000
Thiacloprid	77.0	1484.0	500.5	17 808.0	2226.0	2968.0	17 320 000.0	3 100 000.0	1 000
Year 2020									
Tau-fluvalinate	1.0	19.8	6.5	237.6	1.5	39.6	12 600 000.0	LC50 unknown	53 000
Thiacloprid	3.0	101.0	19.5	1212.0	4.5	202.0	17 320 000.0	LC50 unknown	14 000
HBC	1.0	1.0	6.5	12.0	1.5	2.0	LD50 unknown	LC50 unknown	unknown

The results in 2019 are semiquantitative (LOQ = 10 µg/kg). The pollen consumption is calculated based on data given by the European Safety Authority (2013). The LD50 values describe oral acute toxicity for adult honeybees; exposure level where half of the tested population dies. The LC50 value describes oral 10 days chronic toxicity for adult honeybees; exposure level where half of the tested population dies. The LD50 and LC50 values are reported in European Food Safety Authority’s Conclusion on Pesticide Peer Review (European Food Safety Authority 2019, 2018, 2014, 2010a, 2010b)

^*^The value is calculated by dividing the LD50 value by the highest exposure of a nurse bee. The count is rounded up to the nearest thousand

In 2020, when all the hives where next to oilseed rape fields, tau-fluvalinate was found in eight out of 41 analyzed pollen samples: the residue levels ranging from ≤ 1 to 19.8 µg/kg (Table 3, Table 4 in the Appendix). We did not establish any statistically significant difference between pollen sampled from beehives next to pesticide treated fields and untreated control fields. In nectar samples, none of the analyzed active substances was found. Esfenvalerate was not found in the pollen either. Besides tau-fluvalinate, two other active substances were found in the pollen: thiacloprid and hexachlorobenzene (HBC). HBC is an old pesticide, the use of which the Stockholm Convention on Persistent Organic Pollutants banned in 2001. Neither of the active substances that the fields were treated with before the bee hives were placed on the field margins, flypyradifurone and deltamethrin, were found in the pollen.

None of the active substances found in the pollen exceeded the oral acute toxicity to honeybees. However, due to lack of chronic toxicity data for most of the active substances, the chronic toxicity was evaluated only for two pesticide compounds (indoxacarb and thiacloprid), and their residues found in our study did not exceed the chronic toxicity to honeybees (Table 3). Results of all analyzed samples are given in Tables 4 and 5 in the Appendix.

### Oilseed and caraway pollen identification

In 2019, the honeybees collected mostly pollen from *Brassicaceae* genus which was assumed to be oilseed rape or turnip rape pollen and *Apiaceae* pollen that was assumed to be caraway pollen based on the color and the microscope identification. (Tables 4 and 5 in the Appendix). The next day, after the lambda-cyhalothrin treatment, the pollen sampled from the two hives next to the winter oilseed rape field contained 59% and 75% oilseed pollen, whereas the pollen samples from the hives next to the spring turnip rape field contained 97% and 85% oilseed pollen. The spring turnip rape field was not flowering during the sampling. Respectively, the pollen sampled from the two hives next to the caraway fields contained 14.7% and 5.6% caraway pollen in field 2 and 0% and 8% caraway pollen in field 3. In 2020, based on the amount of the yellow pollen pellets in the samples, the honeybees collected less oilseed pollen than in the previous year. After the last pesticide treatment, the oilseed pollen content in the sampled pollen ranged between 0 and 23%. The oilseed pollen concentration did not correlate with the tau-fluvalinate or thiacloprid residue levels in 2020. Although the used methods (light microscope and pollen color identification) did not give precise information on plant species composition, they gave an approximate picture of the proportion of caraway and oilseeds in the pollen samples.

## Discussion

Our study is one of the few providing knowledge on the protectiveness of the EU Regulation (EC) No. 1107/2009 to pesticide exposure of honeybees, as well as on the field-realistic levels of more than 100 pesticide active substances in honeybee-collected pollen. We found that the residue levels of all studied active substances remained clearly under the oral acute toxicity for honeybees, indicating that the implementation of the EU Regulation protected the honeybees from acute lethal doses of the studied pesticides in the study years. However, information on the levels of oral chronic toxicity for honeybees was available only for indoxacarb and thiacloprid, in the EFSA peer review of the pesticide risk assessment. Thus, the question remained unanswered as to whether the use of other active substances detected in the study caused chronic lethality or poisoning, with effects on colony survival and development. We did not find any residues in the nectar, which may be explained by the rainy weather and the sampling method. The rainy weather conditions decreased the honeybees’ foraging activities, and the sampling method left it uncertain as to whether we sampled freshly collected nectar. Furthermore, in contrast to our expectations, we did not establish a difference in pesticide residues between the beehives located on the field margins of oilseed rape fields treated with esfenvalerate and tau-fluvalinate and the control beehives located next to untreated oilseed rape fields. The lack of statistical difference between the location may be explained by the small sample size together with the large share of samples where no pesticide residues were detected.

We detected several pesticide active substances in the pollen, of which the thiacloprid was most alarming. The maximum residue level we found, 1484 µg/kg, was high compared with previous studies in the EU, where the maximum levels of thiacloprid in honeybee pollen ranged between 133.05 and 1000.2 µg/kg (Beyer et al. 2018; Böhme et al. 2018; Ketola et al. 2015; Pohorecka et al. 2012). Beyer et al. (2018) reported that no bee hive with thiacloprid residues of more than 23 µg/kg in pollen survived over the 3-year study period. Converting 23 µg/kg to thiacloprid oral exposure per adult bee (12 mg pollen/day) is 0.00028 µg/bee – 64 times less than the thiacloprid exposure of an adult honeybee in our study (0.018 µg/bee). In the study of Beyer et al. (2018), the honeybees were also exposed to several active substances other than thiacloprid that may have had synergistic effects on the colony survival. Brandt et al. (2016) studied the effects of field-realistic oral exposure of adult honeybees to thiacloprid, using the exposure of 498 μg/kg and 240 µg/kg detected in the pollen by Rosenkranz et al. (2014) and found that the exposure weakened the immunocompetence of the bees.

In 2019, we studied the pesticide residues only from pollen, although the diet of an adult honeybee mainly consists of plant nectar (European Food Safety Authority, 2013). In the previous study in Finland, Ketola et al. (2015) reported maximum thiacloprid residues of 130 µg/kg in honeybee-collected nectar; the calculated daily thiacloprid consumption in nectar being 0.05 µg for forager bees, 0.02 µg for nurse bees and 0.03 µg for larvae (assuming that the nectar contained 30% sugar (European Food Safety Authority, 2013)). If the residue levels in nectar in 2019 were anywhere near the residue levels of the previous study, the total thiacloprid oral exposure in 2019 was twice as high for a nurse bee and ten times higher for a larva as reported in this study based on the pollen consumption. Due to the high thiacloprid level in the pollen found in our study compared with that reported in the existing literature, we interviewed the farmers cultivating oilseeds, the only likely crop treated with thiacloprid at that time of the growing season, within a radius of 2 km of the beehive where we sampled the maximum thiacloprid residue level. Based on the interviews, we found one field located 1400 m from the experimental field where the farmer had used thiacloprid, according to authorization of the commercial pesticide product, 5 days before the start of our pollen sampling. The thiacloprid residue levels in the pollen samples declined substantially every day, raising a question as to how dramatically higher the thiacloprid level had been 5 days earlier when the farmer had used thiacloprid. The EU banned the use of thiacloprid in open fields after 2020, mainly because of its impact on groundwater and human health (European Food Safety Authority, 2019). According to EFSA, the risks for honeybees were low, though an indication existed of sublethal effects and synergistic effects with other chemical or biological stressors (European Food Safety Authority, 2019).

The residue levels of the three active substances (lamda-cyhalothrin in 2019, tau-fluvalinate in 2020, and esfenvalerate in 2020) that we treated our experimental fields with remained notably below the acute toxicity level for honeybees. The sublethal and chronic effects of the active substances, however, are less studied than those of neonicotinoids such as thiacloprid (Zioga et al. 2020; Benuszak et al. 2017). In the pollen samples, we found residues of lambda-cyhalothrin and tau-fluvalinate, but not of the third active substance used in the experimental fields, esfenvalerate. We did not find any other study that, using a similar methodology than ours, reports lambda-cyhalothrin levels in honeybee-collected pollen and allows a comparison with the levels found in our study. Liao et al. (2018) found lambda-cyhalothrin shortened the honeybee lifetime and memory, but the exposure levels in the study were notably higher than the levels found in our research. The residue levels of tau-fluvalinate were similar to the maximum level of 10 µg/kg in pollen reported by Böhme et al. (2018), but substantially lower than the maximum level of 2670 µg/kg reported by Mullin et al. (2010) and 340 µg/kg reported by Chauzat et al. (2006). The previous Finnish study by Ketola et al. (2015) reported much higher tau-fluvalinate residue levels in beebread, 250 µg/kg, than we found in our study of the pollen. Interestingly, tau-fluvalinate, in addition to being used in plant protection, is used in beekeeping to control the parasitic varroa mite in beehives. Erban et al. (2019) studied tau-fluvalinate treated beehives and noticed that even the detected maximum contact exposure of 0.0071 µg/bee, which was considerably higher than the maximum exposure of 0.00024 µg/bee in our study, did not have a negative effect on the bee colonies. Hence, tau-fluvalinate residues found in our study are not expected to have negative effects on the colony. In our study, we did not control the varroa mite with tau-fluvalinate, and thus, the tau-fluvalinate residues originated from the agricultural area around the beehives. Besides thiacloprid and the two active substances used in the field experiments (lamda-cyhalothrin and tau-fluvalinate), we found indoxacarb, azoxystrobin, tebuconazole, and HCB in the pollen; the residue levels staying at the same level or lower than the limited information contained in the literature (Beyer et al. 2018; Niell et al. 2015; Pohorecka et al. 2012; Böhme et al. 2018).

Overall, we found it very difficult to access all the relevant studies behind the risk assessment of specific active substances, and we often had to rely on the conclusion made by the EFSA. We do not doubt that the necessary data are available, but gathering the scattered data is extremely challenging, leaving room for mistakes and not encouraging the examination of the risk assessment process in relation to one’s own study or other literature. Based on the EFSA conclusions, in semi-field and field studies, the pesticide residues in honeybee matrices are often not studied, but only the applied pesticide dose (active substance/ha or ml product/ha) is reported. We argue that the transparency and the usability of the EU pesticide risk assessment would be greatly enhanced by reporting the exposure levels of honeybees to residue levels in honeybee matrices always when semi-field and field studies are performed. Information regarding the pesticide residue levels would give the global pollinator research not only an additional reference value, indicating acceptable or unacceptable pesticide residue levels in honeybee matrices, but also would increase the knowledge on field-realistic pesticide residue levels. Likewise, a better knowledge on the chronic and sublethal effects of the pesticides on honeybees would enhance the comprehensive understanding of the effects of the field-realistic pesticide residue levels. The current limited knowledge on sublethal effects of the pesticide active substances found in honeybee matrices in our research obliged us mainly to concentrate on lethal doses and to consider honeybees as binary organisms: living or dead. Honeybees, however, are complex organisms, utilizing several cognitive skills, including spatial cognition (Najera & Jander 2012) and metacognition (Perry & Barron 2013), that the sublethal exposure of pesticides may disrupt (Chmiel et al. 2020). Since 2016, the EU risk assessment process requires studies on chronic and sublethal effects of pesticide active substances on honeybees when a novel active substance is approved or when an already approved one is renewed (EU Regulations (EC) 283/2013/EU and 284/2013/EU). However, semi-field and field studies are not required for all pesticide active substance, but only if the toxicity studies and predicted environmental concentrations indicate a high risk for honeybees (European Food Safety Authority, 2013). Better knowledge on sublethal effects of field-realistic pesticide residues on honeybees is crucial for the protection of the species.

Studies on the pesticide exposure of honeybees have used a various sampling methods and matrices, as described by Benuszak et al. (2016). In a recent review article, Zioga et al. (2020) favored studies where nectar and pollen are sampled directly from the plant flower because this data describe the pesticide exposure of individual insects, including wild pollinators, foraging on a specific plant. In our study, we did not take samples directly from the flowers but instead sampled honeybee-collected pollen and nectar, because we found that those matrices better described the exposure of honeybees. If we had sampled pollen and nectar from the crop plants (oilseed rape in 2019 and 2020, turnip rape in 2019, and caraway in 2019), instead of the beehives, the residues had been most likely higher especially in 2020. In 2020, the pollen color identification showed that the bees had mainly foraged in other plant species than oilseed rape (most of the pollen pellets were not yellow color). However, the pesticide exposure of wild pollinator species, for example, solitary bee species that forage in a relatively narrow area (Gathmann & Tscharntke 2002; Gruber et al. 2011), may differ from the exposure of honeybees reported in our study.

The strength of our study is that it provides information on actual pesticide exposure of honeybees under field-realistic conditions, when pesticides are used according to the EU legislation and its national implementation. Furthermore, instead of focusing on individual active substances, we analyzed a broad spectrum of pesticides. Treating the experimental fields with the three studied active substances (lambda-cyhalothrin, esfenvalerate, and tau-fluvalinate), according to the label instructions of the commercial products, provides the national authorities with valuable and specific information on how accurate their pesticide risk assessment is. Few limitations must be considered while interpreting the results of this study. Firstly, the number of study years and locations is not extensive enough to give a comprehensive picture of the annual and geographical variation of the residues in the honeybee matrices. Secondly, we took the nectar samples inside the beehives; a sampling method that always leaves it uncertain as to whether we sampled freshly collected nectar, or misinterpreted the nectar storage of the honeybees, and sampled nectar the honeybees had collected before the pesticide treatments. Lastly, we studied only the oral pesticide exposure of honeybees via pollen and nectar and did not take into account the other recognized exposure routes, such as contact exposure via spray drift or contaminated pollen and nectar, and contamination via soil and water (Benuszak et al. 2017).

## Conclusions

Our study fills an important knowledge gap by providing information on field-realistic levels of pesticide residues in honeybee-collected pollen In light of current research, the implementation of the EU Regulation ((EC) No. 1107/2009) protected honeybees from hazardous oral exposure of the three studied active substances, esfenvalerate, tau-fluvalinate and lambda-cyhalothrin, in the study years and environmental conditions. The high residue levels of the neonicotinoid thiacloprid, however, show the need for constant pesticide residue monitoring in the environment. To have a stronger interface between the EU risk assessment process and the studies conducted by academia outside the process, the EU risk assessment (the data on the effects of the active substances provided by companies) should include and provide an easy access to more studies reporting pesticide residues in honeybee matrices. Multi-year studies on pesticide residues in several matrices in different regions and better knowledge on pesticide chronic toxicity are needed to thoroughly assess the protectiveness of the EU pesticide legislation.

## Data Availability

The datasets used and/or analyzed during the current study are available from the corresponding author on reasonable request.

## Appendix

**Table 4:** Sampling date, sampling data and residue data in 2020 in oilseed rape fields

Field	Treatment	Pesticide application	Sampling date	Time the trap collected pollen (h)	Mean temperature of the sampling day (° C)	Precipitation of the sampling day (mm)	Weight of the collected pollen (g)	Proportion of yellow pollen (%)*	Tau-fluvalinate (µg/kg)	HBC (µg/kg)	Thiacloprid (µg/kg)
1	Treated	None	27/06	8.25	22.7	0.0	21.0	7	0	0	0
1	Treated	Night between 27/06–28/06 (esfenvalerate)	28/06	20	22.3	0.0	38.8	15	0	0	0
1	Treated	05/07 (tau-fluvalinate)	05/07	2.5	14.6	14.8	16.9	13	≤ 3	≤ 1	33.0
1	Treated	05/07 (tau-fluvalinate)	06/07	7.5	14.9	7.1	12.9	1	≤ 1	0	0
1	Treated	10/07 (tau-fluvalinate)	10/07	6.25	11.9	23.5	18.6		5.4	0	28.0
2	Control		28/06	20	22.3	0.0	12.3	9	0	0	0
2	Control		05/07	6	14.6	14.8	31.7	20	0	0	101.0
2	Control		06/07	6	14.9	7.1	31.7	20	0	0	81.0
2	Control		10/07	7.25	11.9	23.5	4.8	23	0	≤ 1	99.0
3	Treated	None	27/06	11.25	22.7	0.0	8.0	24	0	0	0
3	Treated	Night between 27/06–28/06 (esfenvalerate)	28/06	19	22.3	0.0	11.3	21	0	0	0
3	Treated	05/07 (tau-fluvalinate)	05/07	2	14.6	14.8	1.8	12	0	0	≤ 3
3	Treated	05/07 (tau-fluvalinate)	06/07	6	14.9	7.1	5.9	12	0	0	4.0
3	Treated	10/07 (tau-fluvalinate)	10/07	8.75	11.9	23.5			0	0	31.0
4	Treated	None	27/06	10.5	22.7	0.0	3.3	6	0	0	0
4	Treated	Night between 27/06–28/06 (esfenvalerate)	28/06	19	22.3	0.0	12.3	38	0	0	0
4	Treated	05/07 ((tau-fluvalinate)	05/07	2.25	14.6	14.8	5.1	1	≤ 3	≤ 1	≤ 3
4	Treated	05/07 (tau-fluvalinate)	06/07	6	14.9	7.1	11.6	0	0	0	0
4	Treated	10/07 (tau-fluvalinate)	10/07	5.25	11.9	23.5	8.6	16	19.8	0	14.0
5	Control		28/06	23.5	22.3	0.0	7.9	3	0	0	0
5	Control		05/07	2.5	14.6	14.8	2.5	3	0	≤ 1	0
5	Control		06/07	6.75	14.9	7.1	4.5	0	0	≤ 1	0
5	Control		10/07	7.25	11.9	23.5	10.7	10	0	0	0
6	Control		28/06	23.5	22.3	0.0	22.0	0	0	0	0
6	Control		05/07	3.75	14.6	14.8	8.6	41	0	0	0
6	Control		06/07	5.75	14.9	7.1	0.6	0	0	≤ 1	0
6	Control		10/07	7.5	11.9	23.5	2.8		0	0	0
7	Control		28/06	23.5	22.3	0.0	33.4	8	0	0	0
7	Control		05/07	4.5	14.6	14.8	23.9	13	≤ 3	0	0
7	Control		06/07	6	14.9	7.1	5.4	25	0	0	0
7	Control		10/07	7.5	11.9	23.5	9.5	4	6	0	0
8	Treated	None	27/06	6.5	22.7	0.0	2.8	2	0	0	0
8	Treated	Night between 27/06–28/06 (esfenvalerate)	28/06	23.5	22.3	0.0	16.7	17	0	0	0
8	Treated	05/07 (tau-fluvalinate)	05/07	3.25	14.6	14.8	6.4	7	0	0	0
8	Treated	05/07 (tau-fluvalinate)	06/07	5.25	14.9	7.1	2.0	10	0	≤ 1	0
8	Treated	10/07 (tau-fluvalinate)	10/07	7	11.9	23.5	1.8		0	0	0
9	Treated	None	27/06	6.5	22.7	0.0	8.8	2	0	0	0
9	Treated	Night between 27/06–28/06 (esfenvalerate)	28/06	23.75	22.3	0.0	17.4	7	0	0	0
9	Treated	05/07 (tau-fluvalinate)	05/07	4.25	14.6	14.8	14.8	2	0	0	0
9	Treated	05/07 (tau-fluvalinate)	06/07	4.5	14.9	7.1	10.2	0	0	0	0
9	Treated	10/07 (tau-fluvalinate)	10/07	7.25	11.9	23.5	4.7	4	10.5	0	0

*Yellow color indicates rape seed or turnip rape pollen.

The pollen traps were installed immediately after the pesticide application.

**Table 5:** Sampling date, sampling data, and residue data in pollen sampled in 2019

Field	Crop	Lambda-cyhalothrin application	Sampling date	Mean temperature of the sampling day (° C)	Precipitation of the sampling day (mm)	Percentage of crop plant pollen *Brassicacea (*oilseed rape and turnip rape*)* or *Apiaceae* (caraway)		Hive from which the pesticide residues are analyzed	Lambda-cyhalothrin (µg/kg)	Thiacloprid (µg/kg)	Tebuconazole (µg/kg)	Indoxacarb (µg/kg)	Azoxystrobin (µg/kg)
						Hive 1	Hive 2						
1	Oilseed rape	20/05	21/05	18.9	1.1	75.0	56.6	Pooled sample	≈5.2	0	0	0	0
1	Oilseed rape	20/05	22/05	17.4	4.2	79.8	78.3	Pooled sample	0	0	0	0	0
1	Oilseed rape	20/05	23/05	14.1	2.7	78.5	68.4	Hive 2	0	0	0	0	0
1	Oilseed rape	20/05	04/06	17.3	0.0	5.3	15.7	Pooled sample	0	0	0	0	0
1	Oilseed rape	20/05	05/06	19.9	0.0	6.3	5.7	Pooled sample	0	0	0	0	0
1	Oilseed rape	20/05	06/06	23.7	0.0	28	22.7	Pooled sample	0	0	0	0	0
2	Caraway	03/06	04/06	17.3	0.0	14.7	5.6	Pooled sample	≈ 7.8	0	0	0	≈ 3.3
2	Caraway	03/06	05/06	19.9	0.0	8.9	4.9	Hive 2	0	0	0	0	0
2	Caraway	03/06	06/06	23.7	0.0	3.7	3.2	Pooled sample	0	0	0	0	0
2	Caraway	03/06	10/06	15.2	0.0	24.6	12.1	Hive 2	0	0	0	0	≈ 3.8
2	Caraway	03/06	11/06	17.0	0.0	36.9	17.4	Hive 2	0	0	0	0	0
2	Caraway	03/06	12/06	13.9	0.0	20.3	13.6	Pooled sample	0	0	0	0	0
3	Caraway	03/06–04/06	04/06	17.3	0.0	0.0	8.6	Pooled sample	≈ 3.2	0	≈ 0.3	0	0
3	Caraway	03/06–04/06	05/06	19.9	0.0	0.0	0.0	Pooled sample	0	0	0	0	0
3	Caraway	03/06–04/06	06/06	23.7	0.0	35.6	0	Pooled sample	0	0	0	0	0
3	Caraway	03/06–04/06	10/06	15.2	0.0	13.0	12.7	Pooled sample	0	0	0	0	0
3	Caraway	03/06–04/06	11/06	17.0	0.0	14.0	12.6	Pooled sample	0	0	0	0	0
3	Caraway	03/06–04/06	12/06	13.9	0.0	15.9	0.0	Pooled sample	0	0	0	0	0
4	Turnip rape	01/06 and 25/06	25/06	14.3	1.4	96.9	84.6	Pooled sample	0	1484.0	0	≈ 2.5	0
4	Turnip rape	01/06 and 25/06	26/06	14.2	3.4	96.2	68.9	Pooled sample	0	698.0	0	0	0
4	Turnip rape	01/06 and 25/06	28/06	14.1	0.0	61.7	53.9	Pooled sample	0	247.0	0	0	0
4	Turnip rape	01/06 and 25/06	03/07	12.5	0.0	73.2	27.3	Pooled sample	0	77.0	0	0	0
4	Turnip rape	01/06 and 25/06	12/07	14.7	0.0	49.3	43.2	Hive 2	0	0	≈ 1.3	0	0

Concentrations are semi-quantitative (LOQ = 10 µg/kg).

**Table 6:** Analyzed active substances and (limit of quantification LOQ) in nectar and pollen in 2020

Active substance	GC or LC	m/z perursor > product	LOQ (µg/kg)	
		Quant. ion	Nectar	Pollen
4,4'- Methoxychlor	GC	227.01 > 169.01	1	4
Acetamiprid	LC	223.20 > 126.10	1	4
Aldrin	GC	292.90 > 185.93	1	4
Alpha-endosulfan	GC	271.88 > 236.89	1	4
alpha-HCH	GC	216.89 > 180.91	1	4
Amiratz	LC	294.10 > 162.90	1	4
Atzinphos-ethyl	GC	160.02 > 132.01	10	30
Atzinphos-methyl	GC	160.02 > 132.01	10	30
Azoxystrobin	LC	403.50 > 372.20	1	4
beta-endosulfan	GC	242.89 > 207.91	1	4
beta-HCH	GC	216.89 > 180.91	1	4
Bifenthrin	GC	181.05 > 166.05	1	4
Bixafen	GC	159.00 > 139.11	1	4
Boscalid	GC	342.03 > 140.01	1	4
Bromopropylate	GC	342.96 > 184.98	1	4
Buprofezin	GC	105.00 > 77.00	1	4
Carbendazim	LC	191.50 > 160.10	1	4
Carbetamide	LC	237.00 > 192.50	1	4
Chinomethionat	GC	233.99 > 205.99	1	4
Chlordimeform	LC	197.20 > 117.15	10	30
Chlorfenvinphos	GC	266.90 > 159.10	1	4
Chlorobenzylate	GC	139.00 > 111.00	1	4
Chlorpropham	GC	213.06 > 171.04	1	4
Chlorpyrifos-methyl	GC	285.91 > 270.91	1	4
Chlorpyriphos	GC	313.93 > 257.95	1	4
cis-chlordane	GC	372.81 > 265.87	1	4
Clothianide	LC	250.20 > 169.00	1	4
Coumaphos	GC	226.01 > 163.01	10	30
Cyfluthrin	GC	163.02 > 127.02	10	30
Cymiazole	LC	219.00 > 171.00	1	4
Cypermethrin	GC	163.02 > 127.02	10	30
Cyproconazole	GC	222.09 > 125.05	1	4
Cyprodinil	LC	225.70 > 93.10	1	4
DEET	GC	119.10 > 91.00	10	30
Deltametrin	GC	250.99 > 171.99	1	4
Diazinon	LC	305.10 > 168.50	10	30
Dieldrin	GC	276.91 > 240.92	1	4
Difenoconazole	GC	225.70 > 93.10	10	30
Dimetoat	LC	229.90 > 199.10	1	4
Dimetomorph	LC	387.60 > 301.10	1	4
Dimoxystrobin	LC	326.70 > 205.20	1	4
Endosulfane-sulfate	GC	273.88 > 238.89	1	4
Endrin	GC	280.91 > 244.92	10	30
Epoxiconazole	GC	192.04 > 111.02	10	30
Esfenvalerate	GC	419.13 > 225.07	10	30
Ethoprophos	LC	243.00 > 130.50	1	4
Etofenprox	GC	163.09 > 107.06	1	4
Fenhexamid	LC	302.00 > 55.10	10	30
Fenpropidine	LC	273.90 > 147.00	10	30
Fenpropimorph	GC	128.11 > 70.06	1	4
Fenvalerate	GC	419.13 > 225.07	10	30
Flupyradifuron	LC	289.00 > 126.10	10	30
Fluquinconazole	GC	340.01 > 298.10	1	4
Flusilazole	GC	233.07 > 165.05	1	4
Flutriafol	GC	123.00 > 95.00	1	4
Glyphosate (FMOC)	LC	392.00 > 88.00	10	40
HCB	GC	283.81 > 248.84	0.5	2
Heptachlor exooepoxide	GC	352.83 > 218.88	10	30
Heptachlor	GC	336.84 > 301.85	10	30
Heptachlorendo epoxide	GC	352.83 > 218.88	10	30
Heptenophos	LC	251.10 > 127.10	10	30
Imazalil	LC	297.00 > 159.10	10	30
Imidacloprid	LC	255.90 > 209.20	1	4
Indoxacarb	GC	203.03 > 106.01	10	30
Lambda-cyhalothrin	GC	208.05 > 181.04	1	4
Lindane	GC	216.89 > 180.91	1	4
Malaoxon	GC	195.04 > 167.03	20	30
Malathion	GC	173.02 > 145.02	20	30
Metaflumizone	LC	507.00 > 178.20	1	4
Methidathion	GC	144.98 > 84.99	1	4
Methiocarb	LC	225.80 > 169.20	1	4
Myclobutanil	LC	289.00 > 70.10	1	4
op-DDT	GC	234.97 > 164.98	1	4
Oxyclordane	GC	386.79 > 262.86	1	4
Parathion	GC	291.03 > 137.02	10	30
Parathion-methyl	GC	263.00 > 108.99	1	4
Penconazole	GC	248.06 > 157.04	1	4
Pendimethalin	GC	252.12 > 162.02	1	4
Permethrin	GC	183.04 > 168.03	10	30
Phosalone	LC	367.90 > 182.10	1	4
Pirimicarb	LC	239.20 > 72.00	1	4
Pirimicarb-desmethyl	LC	225.00 > 72.10	1	4
Pirimiphos methyl	LC	306.00 > 164.00	10	30
pp-DDD	GC	234.97 > 164.98	1	4
pp-DDE	GC	245.95 > 175.97	1	4
pp-DDT	GC	234.97 > 164.98	1	4
Procloraz	LC	376.00 > 308.00	1	4
Profenofos	GC	337.00 > 267.00	1	4
Propargite	LC	368.10 > 231.30	1	4
Prothioconazole	LC	344.10 > 325.80	1	4
Pyraclostrobin	LC	387.60 > 194.20	1	4
Pyrazophos	GC	265.06 > 210.05	1	4
Pyrimethanil	GC	198.00 > 183.10	1	4
Resmethrin	GC	171.11 > 128.08	10	30
Spinosad	LC	732.20 > 142.20	10	30
Spiroxamine	LC	297.90 > 144.20	1	4
Tau-fluvalinate	GC	250.06 > 200.05	1	4
Tebuconazole	GC	252.12 > 127.06	1	4
Tebufenozide	LC	351.00 > 149.00	1	4
Tert-butylazine	GC	172.90 > 172.00	1	4
Tetraconazole	GC	336.02 > 218.01	1	4
Thiacloprid	LC	252.70 > 126.10	1	4
Thimetoxam	LC	291.90 > 211.20	1	4
Thiophanate-methyl	LC	343.10 > 151.00	10	30
Trans-chlordane	GC	372.81 > 265.87	1	4
Triadimefon	GC	210.07 > 183.06	1	4
Triazophos	GC	257.05 > 162.03	1	4
Trifloxystrobin	LC	408.60 > 186.20	1	4
Vinclozolin	GC	287.00 > 214.00	1	4

In 2019 the analyzed substances were the same as in 2020, but in 2019, tebufenozide and flupyradifurone were not analyzed. Furthermore, in 2019, the LOQs in pollen were higher than in 2020, being 10–40 µg/kg for all active substances.

**Table 7:** Weather conditions in Jokioinen in 2019 and 2020

**May 2019**							
**Date**	**Temperature**				**Precipitation**		**Relative humidity**
	Effective						
	Mean	temp. sum	Max	Min		Sum	(mean)
	° C	° C	° C	° C	mm	mm	%
1	6.8	41	15.0	− 1.2	4.6	4.6	66
2	2.5	41	5.1	0.3	5.8	10.4	95
3	0.3	41	3.5	− 1.4	0.0	10.4	65
4	1.2	41	5.9	− 3.9	1.2	11.6	76
5	3.9	41	10.0	− 3.4	0.0	11.6	71
6	5.2	41	12.0	− 1.8	0.0	11.6	67
7	6.0	42	11.0	1.3	0.0	11.6	66
8	5.8	43	11.0	1.3	0.0	11.6	62
9	9.2	47	15.3	1.0	0.0	11.6	48
10	8.6	51	12.7	4.3	1.0	12.6	76
11	9.4	55	12.9	7.2	6.7	19.3	94
12	8.6	59	11.8	7.7	0.0	19.3	87
13	6.6	60	10.6	1.2	0.0	19.3	64
14	7.2	63	13.1	− 0.8	0.0	19.3	53
15	8.8	66	16.2	− 1.9	0.0	19.3	56
16	11.5	73	18.7	0.1	0.0	19.3	57
17	14.0	82	21.2	2.9	0.0	19.3	55
18	16.0	93	23.7	3.9	0.0	19.3	50
19	17.7	106	25.3	6.5	0.0	19.3	47
20	19.4	120	26.3	13.7	0.1	19.4	56
21	18.9	134	26.7	13.7	1.1	20.5	70
22	17.4	146	24.7	9.4	4.2	24.7	76
23	14.1	155	20.3	13.3	2.7	27.4	80
24	8.8	159	14.6	6.8	0.8	28.2	90
25	9.7	164	11.4	8.4	8.5	36.7	96
26	8.0	167	10.1	6.7	8.3	45.0	97
27	10.2	172	14.0	7.0	0.1	45.1	88
28	11.3	178	16.3	4.7	5.5	50.6	82
29	10.5	184	17.0	8.9	0.0	50.6	74
30	10.8	190	16.0	3.2	6.0	56.6	50
31	10.8	196	14.0	7.3	0.0	56.6	62
**June 2019**							
**Date**	**Temperature**				**Precipitation**		**Relative humidity**
	Effective						
	Mean	temp. sum	Max	Min		Sum	(mean)
	° C	° C	° C	° C	mm	mm	%
1	11.0	6	17.2	4.6	6.8	6.8	71
2	11.6	13	16.5	8.0	0.0	6.8	75
3	12.7	20	18.6	1.9	0.3	7.1	60
4	17.3	33	21.2	13.0	0.0	7.1	77
5	19.9	48	26.2	11.6	0.0	7.1	69
6	23.7	66	29.8	15.1	0.0	7.1	56
7	23.7	85	28.1	16.8	0.0	7.1	60
8	23.2	103	28.8	16.4	16.8	23.9	66
9	17.5	116	24.6	15.7	0.0	23.9	73
10	15.2	126	19.9	11.5	0.0	23.9	65
11	17.0	138	23.2	8.3	0.0	23.9	59
12	13.9	147	19.5	8.9	0.0	23.9	53
13	14.6	156	19.0	11.0	0.0	23.9	73
14	14.9	166	16.9	13.5	0.3	24.2	96
15	14.8	176	21.0	10.3	0.0	24.2	84
16	17.8	189	24.5	7.9	0.0	24.2	68
17	18.2	202	25.8	9.1	1.5	25.7	67
18	19.2	216	24.5	13.0	0.0	25.7	61
19	18.8	230	24.0	13.9	0.0	25.7	68
20	21.2	246	27.2	12.3	0.0	25.7	60
21	20.5	262	25.7	16.8	0.6	26.3	66
22	15.1	272	20.7	12.2	0.0	26.3	67
23	15.1	282	21.7	7.0	0.0	26.3	59
24	16.0	293	20.8	8.9	0.0	26.3	55
25	14.3	302	21.6	5.7	1.4	27.7	68
26	14.2	311	18.4	11.2	3.4	31.1	82
27	14.5	321	19.4	12.5	0.0	31.1	75
28	14.1	330	19.8	8.5	0.0	31.1	56
29	14.8	340	20.6	7.4	0.0	31.1	65
30	17.5	352	22.4	11.9	0.0	31.1	60
**July 2019**							
**Date**	**Temperature**				**Precipitation**		**Relative humidity**
	Effective						
	Mean	temp. sum	Max	Min		Sum	(mean)
	° C	° C	° C	° C	mm	mm	%
1	18.1	561	22.7	15.4	3.8	3.8	68
2	13.8	570	17.8	12.7	3.7	7.5	86
3	12.5	577	18.0	7.1	0.0	7.5	57
4	11.8	584	16.6	5.0	9.8	17.3	65
5	11.1	590	14.9	6.6	29.7	47.0	90
6	13.2	598	19.6	9.2	3.1	50.1	82
7	12.5	606	18.9	6.1	8.6	58.7	90
8	14.0	615	17.8	11.4	1.8	60.5	89
9	11.8	622	13.5	10.0	0.0	60.5	84
10	15.7	632	21.1	9.1	0.0	60.5	59
11	12.9	640	18.0	11.9	0.1	60.6	71
12	14.7	650	19.7	7.7	0.0	60.6	60
13	14.8	660	20.4	8.7	0.0	60.6	59
14	14.0	669	19.3	7.1	0.0	60.6	67
15	14.5	678	20.5	6.3	7.3	67.9	73
16	14.7	688	19.9	12.1	1.7	69.6	88
17	16.2	699	22.9	10.2	0.0	69.6	77
18	16.6	711	22.5	8.3	0.0	69.6	68
19	17.5	723	23.2	8.6	0.0	69.6	62
20	19.0	737	25.6	9.2	0.0	69.6	60
21	20.0	752	26.8	9.8	0.0	69.6	61
22	18.7	766	23.8	11.9	0.6	70.2	79
23	18.5	779	25.1	12.7	2.5	72.7	83
24	20.5	795	27.1	11.5	0.0	72.7	69
25	21.8	812	28.3	14.3	0.0	72.7	65
26	21.1	828	27.3	12.0	0.0	72.7	71
27	23.4	846	30.8	13.7	0.0	72.7	65
28	24.5	866	31.4	15.4	0.0	72.7	57
29	18.7	879	26.0	14.9	0.0	72.7	57
30	13.0	887	18.4	10.5	0.3	73.0	57
31	14.0	896	20.3	9.1	0.0	73.0	63
**June 2020**							
**Date**	**Temperature**				**Precipitation**		**Relative humidity**
	Effective						
	Mean	temp. sum	Max	Min		Sum	(mean)
	° C	° C	° C	° C	mm	mm	%
1	17.1	12	24.1	9.9	0.0	0.0	58
2	13.7	21	19.3	5.3	0.0	0.0	53
3	13.7	30	19.5	4.9	0.0	0.0	51
4	14.6	39	21.7	5.4	1.5	1.5	59
5	13.8	48	17.9	11.2	1.0	2.5	83
6	12.5	55	17.3	10.2	6.9	9.4	84
7	14.4	65	19.5	9.8	0.0	9.4	61
8	14.0	74	19.4	5.7	0.3	9.7	67
9	16.5	85	21.6	11.7	0.0	9.7	61
10	17.8	98	23.2	9.2	0.0	9.7	47
11	18.8	112	24.0	11.9	0.0	9.7	47
12	18.1	125	23.5	8.4	0.0	9.7	52
13	18.6	139	25.4	7.8	0.0	9.7	50
14	18.3	152	24.3	8.3	0.0	9.7	48
15	19.5	166	26.4	7.6	0.0	9.7	54
16	19.7	181	25.2	14.5	2.1	11.8	81
17	20.2	196	26.2	15.2	0.0	11.8	67
18	18.4	210	24.3	12.7	0.0	11.8	68
19	20.5	225	26.4	10.8	0.0	11.8	64
20	21.0	241	25.7	15.3	0.0	11.8	57
21	18.1	254	23.1	11.5	0.0	11.8	58
22	18.2	268	24.1	8.2	0.0	11.8	53
23	20.7	283	28.0	9.7	0.0	11.8	52
24	22.6	301	29.5	11.9	0.0	11.8	46
25	22.6	318	30.6	11.8	0.0	11.8	50
26	21.6	335	28.8	13.2	0.0	11.8	53
27	22.7	353	30.6	11.8	0.0	11.8	54
28	22.3	370	27.8	14.6	0.0	11.8	62
29	17.9	383	23.1	15.3	4.2	16.0	74
30	14.8	393	16.2	13.4	16.2	32.2	94
**July 2020**							
**Date**	**Temperature**				**Precipitation**		**Relative** **humidity**
	Effective						
	Mean	temp. sum	Max	Min		Sum	(mean)
	° C	° C	° C	° C	mm	mm	%
1	14.0	520	18.1	12.8	1.3	1.3	87
2	12.8	528	18.8	5.7	5.3	6.6	83
3	15.8	539	19.0	12.4	0.0	6.6	71
4	13.8	548	16.2	11.0	13.6	20.2	91
5	14.6	557	17.4	11.3	14.8	35.0	85
6	14.9	567	17.1	13.5	7.1	42.1	76
7	13.1	575	16.2	12.3	6.1	48.2	90
8	13.0	583	17.5	9.6	2.9	51.1	84
9	11.7	590	16.1	8.5	2.5	53.6	84
10	11.9	597	16.7	6.5	23.5	77.1	93
11	11.8	604	15.0	9.3	13.6	90.7	94
12	12.9	612	18.2	6.3	7.7	98.4	89
13	12.5	619	18.7	7.3	2.7	101.1	87
14	13.9	628	19.9	5.3	0.0	101.1	75
15	16.0	639	22.4	7.8	0.0	101.1	70
16	17.9	652	24.3	9.5	0.0	101.1	70
17	19.6	667	25.3	10.7	0.0	101.1	68
18	19.6	681	25.0	13.7	0.0	101.1	71
19	19.6	696	25.4	12.9	0.0	101.1	74
20	19.7	710	24.8	14.5	0.6	101.7	78
21	17.4	723	21.7	13.6	2.5	104.2	82
22	13.2	731	17.5	10.5	1.1	105.3	80
23	12.2	738	13.6	10.7	5.5	110.8	83
24	10.5	744	12.1	9.3	6.2	117.0	95
25	13.9	753	17.4	10.1	0.0	117.0	86
26	16.3	764	21.8	7.6	0.0	117.0	71
27	18.0	777	22.5	12.3	0.1	117.1	72
28	17.5	789	21.1	14.7	0.2	117.3	88
29	15.2	800	18.8	14.2	13.8	131.1	93
30	14.8	809	19.2	9.6	3.4	134.5	89
31	16.3	821	21.8	10.6	0.0	134.5	77

Data from the observatory of Jokioinen (location 60.81402°N, 23.49829°E according to map datum WGS 84, altitude 104 m). Data source: Finnish Meteorological Institute.
